# Drivers and barriers of seasonal influenza vaccination 2015/16 & 2019/20 to 2022/23 - a survey on why most Norwegians don’t get the flu vaccine

**DOI:** 10.1186/s12889-024-20157-w

**Published:** 2024-10-02

**Authors:** Birgitte Klüwer, Kjersti Margrethe Rydland, Svenn-Erik Mamelund, Rebecca Nybru Gleditsch

**Affiliations:** 1 Division of Infection Control, Norwegian Institute of Public Health, Skøyen, PO Box 222, Oslo, N-0213 Norway; 2 Centre for Research on Pandemics & Society (PANSOC), Oslo Metropolitan University, Senter for velferds- og arbeidslivsforskning, OsloMet – storbyuniversitetet, Postboks 4, St. Olavs plass, Oslo, 0130 Norway; 3 The Fafo Institute for Labour and Social Research, Tøyen, PO Box 2947, Oslo, N-0608 Norway

**Keywords:** Influenza vaccines, Vaccination hesitancy, Vaccination coverage, Health Knowledge, attitudes, Practice, Public Health, Health personnel, Immunization programmes, Surveys and questionnaires, Facilitators, Barriers

## Abstract

**Background:**

This study aimed to explore the reasons adults in the general population, influenza risk groups (RGs) and health care workers (HCWs) in Norway give for their vaccination choices and whether these reasons vary between groups or over time in order to further improve influenza vaccination coverage.

**Methods:**

Respondents of a nationally representative telephone survey conducted by Statistics Norway were asked “What was the most important reason why you did/did not get vaccinated?”. The question on influenza non-vaccination was included in 2016 and in 2020 to 2023 and the question on influenza vaccination in 2021 to 2023.

**Results:**

The study included 9 705 individuals aged 18–79 years. Influenza vaccination coverage in the RGs increased from 20.6% in 2016 to 63.1% in 2022, before a reduction to 58.3% in 2023. Common reasons for non-vaccination were similar in all groups. The most cited reasons were “no need” for the vaccine and “no specific reason”, followed by “not recommended/offered the vaccine”, “worry about side effects” and “vaccine refusal”. The most frequent reasons for vaccination among the general population and RGs were protection against influenza and belonging to a RG, while the most frequent responses among HCWs were being offered the vaccine at work/work in health care, followed by a desire for protection against influenza. Receiving a vaccine recommendation from a health professional was mentioned in all groups. We also observed that the proportion reporting “no need” for the vaccine decreased over time, especially among HCWs, and that the proportions reporting vaccine refusal and worry about side effects as reasons for non-vaccination were temporarily reduced during the COVID-19 pandemic.

**Conclusions:**

The general population and RGs cite protection against influenza as their primary incentive for vaccination, while HCWs mainly refer to their professional role or workplace vaccination. For non-vaccination we see a similar pattern in all groups, with “no need” and “no specific reason” as the main reasons. Of note, worry about side effects and vaccine refusal is as frequent among HCWs as in other groups. Continued efforts to maintain and increase vaccine confidence are needed.

## Background

Influenza viruses are associated with a substantial disease burden worldwide. Recent global estimates attributes up to 5 million hospitalisations and 650 000 deaths to influenza-associated respiratory disease each year [1–3]. Since the first inactivated influenza vaccine was approved for use in the United States in the 1940s [4] it has become one of the most widely used vaccines, with more than 500 million doses distributed annually [5]. Annual influenza vaccination has been shown to be safe, cost-effective and to reduce risk of severe influenza-related disease and mortality [6]. Both the World Health Organization and the European Union have issued recommendations that member states should aim for an influenza vaccine coverage of at least 75% among both risk groups (RGs) [7] and health care workers (HCWs) [8].

In Norway, influenza vaccines have been in use since they were licensed in Europe in the 1960s, and the vaccine is offered on a voluntary basis [4, 9]. As of 2024, influenza vaccination is recommended annually for RGs (e.g., age 65 years or older, residents in nursing homes, pregnant women, and children and adults with a range of chronic conditions) and HCWs [10]. While there have been variations in the associated cost of influenza vaccines in recent years, with RGs being entitled to free or nearly free vaccinations during the COVID-19 pandemic (influenza seasons 2020/21 to 2021/22), most years the Norwegian population - including RGs - have had to pay to be vaccinated. The cost of vaccination normally ranges from 150 to 500 Norwegian kroner (approximately $15–50 during the study period), depending on place of residence (between municipalities) and vaccine provider (general practitioner (GP), municipality, pharmacy) [11]. HCWs are not included in the Norwegian National Influenza Immunisation Programme, but they are entitled to free vaccination from their employer.

Even though cost has been suggested as a barrier to influenza vaccination [12–15], the estimated number of administered influenza vaccine doses in Norway increased from 436 000 doses in the 2014/15 influenza season to 1 302 000 doses in the 2020/21 influenza season [16]. This corresponds to more than a tripling of the estimated influenza vaccination coverage in the total population from 8.4 to 30.5%. The increase started among the elderly, HCWs and the highly educated, initially resulting in increased educational disparities in vaccination coverage. However, when influenza vaccination was provided for free for RGs during the COVID-19 pandemic, it increased coverage further while the educational disparities diminished. This suggested that free influenza vaccination might be needed to keep socioeconomic differences in vaccination coverage to a minimum [11].

Public opinion also matters in vaccination. It may fluctuate over time and in relation to the severity of recent outbreaks, adverse events reports and media coverage, and it may also vary between vaccines [17]. Studies that have examined attitudes to vaccination in Norway have found high overall confidence in vaccines included in the Norwegian Childhood Immunisation Programme, corresponding to consistently high vaccination coverage over time [18]. However, both vaccination coverage and results from a similar study on confidence in influenza vaccination have indicated a lower confidence in influenza vaccines, both in the general public and in the primary target groups RGs and HCWs [19]. Moreover, while this study found that confidence in influenza vaccines increased over time, it also found that higher educational attainment corresponded to higher confidence throughout the study period from 2017 to 2022, both in the general sample, among the RGs, and among the HCWs [19]. This indicated a need for measures specifically aimed at increasing influenza vaccine knowledge and confidence in groups with lower levels of education.

The aim of the present study was to explore why people accept or reject influenza vaccination in Norway, both among the general population and the target groups. The study period spans influenza seasons before, during and after the COVID-19 pandemic (2015/16 & 2019/20 to 2022/23).

## Methods

### Data source and study sample

The study is based on data from Statistics Norway’s Travel & Vacation survey (T&V-survey), a nationally representative cross-sectional survey that provides quarterly data for official statistics on the travel behaviour of the Norwegian population, as well as other topics [20]. The T&V-survey is conducted as an interviewer-administered, computer-assisted telephone interview (CATI). A new sample of 2000 Norwegians aged 16–79 years are invited to participate each quarter. Stratified random sampling from the National Population Register based on age, sex and county of residence ensure that each quarterly sample mirrors the population in Norway. Informed consent is obtained from each respondent, and the data have been de-identified by Statistics Norway prior to analysis [20].

Questions regarding influenza risk and vaccination status have been included in the T&V-survey twice a year (in the second (Q2) and third (Q3) quarter) since 2015. This study analyses data from all study years that included questions on reasons for influenza vaccination or non-vaccination. Reasons for non-vaccination were included in Q2 2016 and in Q2/Q3 from 2020 to 2023, while reasons for vaccine acceptance were included in Q2/Q3 from 2021 to 2023. In total, 18 000 individuals aged 16–79 years were eligible to participate in the T&V-survey during the study period. 9 806 of these responded, resulting in a response rate of 54.5% (55.7% in Q2 2016; 61.7% in 2020; 53.7% in 2021; 50.1% in 2022, and 52.2% in 2023). After exclusion of 101 respondents with missing information on vaccination status (*n* = 72), chronic conditions related to influenza risk (*n* = 55) and/or status as a HCW (*n* = 54), the final sample consisted of 9 705 individuals.

### Variables

All variables included in this analysis are self-reported, except age, which was obtained from the National Population Register in the sampling process. Vaccination status was established based on the question “Did you get vaccinated against influenza in the course of the last 12 months?”, with response alternatives “yes”, “no”, “do not know” or “do not want to answer”. “Do not know” and “do not want to answer” were coded as missing. Respondents that were 65 years or older, and/or confirmed that they had at least one chronic condition related to increased risk of severe influenza, were coded as belonging to the RGs [10]. Lastly, individuals who answered affirmatively on the question “Do you work in health care and have contact with patients” were categorised as HCWs.

### Reasons for influenza non-vaccination

Questions concerning barriers of influenza vaccination were included for the first time in Q2 of the 2016 T&V-survey. All respondents that reported that they did *not* get the influenza vaccine prior to the 2015/16 influenza season were asked an open-ended question on what they considered their most important reason for non-vaccination. The free-text responses were recorded in full by the interviewers, and later categorised by two of the authors (BK, KMR). Prior to the categorisation process the sample was split into three subsamples, namely respondents that did not belong to any vaccination target group, RGs and HCWs (HCWs reporting chronic conditions were included in both the RG and the HCW subsamples, *n* = 21), to look for similarities and differences in reasons for non-vaccination between those not recommended annual influenza vaccination and the two target groups. Each author then sorted the free-text responses – mainly short statements – into broad themes identified from the data, before coming together to compare their results, discuss any dissimilarities in their individual coding and establish common categories. This resulted in a variable on reasons for influenza non-vaccination with 8 separate response categories, in addition to an “other” category for responses that were either few in number or unclear. The categories for the variable on reasons for non-vaccination, including sample responses for each category, are presented in Table 1. While the final categorisation process was guided in part by the definition of vaccine hesitancy as it was first formulated by the SAGE Working Group on Vaccine Hesitancy and the 3 C’s (complacency, confidence, convenience) model of vaccine hesitancy [21], most of the categories were chosen because of their frequency in one or all subsamples, and each were named for frequently recurring formulations within each category. However, some categories were included despite of few observations in order to estimate their frequency in future surveys, such as “fear of doctor or injections” (Table 1, category 4), or based on the authors’ prior experience regarding common objections from the public or HCWs, such as “prefer natural immunity” (Table 1, category 5).

**Table 1:** Categories of reasons for influenza non-vaccination with free-text sample responses

Category	Sample responses
1. Do not need the vaccine	- “I am healthy and not afraid of getting sick” - “Never had the flu” - “I don’t feel that I am at risk” - “I’m not part of a risk group”
2. No specific reason	- “I haven’t thought about it” - “No specific reason”
3. Not recommended/offered the vaccine	- “Because I have not been told to get the vaccine” - “I did not get an offer from the GP”
4. Fear of doctors or injections	- “Have a fear of doctors” - “I have a fear of injections”
5. Prefer natural immunity	- “I want to get the flu in order to become immune” - “I believe that it’s better for the body to heal itself”
6. Do not believe in the vaccine	- “I don’t think it is effective” - “[The vaccine] is just nonsense”
7. Worry about side effects	- “Fear of side effects” - “I got sick the last time I got vaccinated”
8. Vaccine refuser	- “I don’t want poison in my body” - “I am sceptical of vaccines; I don’t think they are necessary” - “I’m a vaccine denier”
99. Other	- “Allergic” - “I was pregnant” - “Because of the cost of the flu vaccine”

Commonly reported reasons for influenza non-vaccination, with sample responses, based on free-text responses to the question «What was the most important reason why you did not get vaccinated?». Data from Statistics Norway’s T&V-survey, Q2 2016

The question “What was the most important reason why you did not get vaccinated” was repeated in the influenza section of the Q2 and Q3 T&V-survey in 2020, covering the pre-pandemic 2019/20 influenza season, and in 2021 to 2023, covering the influenza seasons during and after the COVID-19 pandemic; 2020/21, 2021/22 and 2022/23. During these four seasons the respondents’ answers were categorized directly by the interviewers during the interview if the answers corresponded to one of the categories presented in Table 1. Answers the interviewers were unsure of, and answers that did not clearly fit any of the predefined categories, were categorized as “other” and recorded in full. As in the original coding process on the 2016-data, the free-text responses of the “other” category were thereafter manually reviewed and coded by three of the authors (BK, KMR, RNG) to assess whether some of the responses coded as “other” should be recoded because they were consistent with already existing categories, or if new categories were needed when these responses were considered. The review was first done independently by each author, and then compared for consistency before final agreement. There were very few discrepancies between the authors in their primary assessment, and these were quickly resolved by discussion. While some free-text answers were assigned to already existing categories during this process, no changes were made to the categories of the variable.

### Reasons for influenza vaccination

A corresponding variable on reasons *for* influenza vaccination was created in collaboration with Statistics Norway in 2020. The question “State the most important reason why you got vaccinated” was thereafter included in Q2 and Q3 of the 2021 to 2023 T&V-surveys, covering influenza seasons 2020/21 to 2022/23 (Table 2). As with the variable on non-vaccination, the respondents gave free-text answers that were either directly categorised by the interviewers according to predefined categories, or they were categorised as “other” and the answer recorded in full for later review by the authors (BK, KMR, RNG). The variable was edited to add a category on media in 2022, to capture whether the massive media coverage and information campaigns on COVID-19 during this period influenced individual vaccine decisions. Two additional categories were then added in 2023 based on the free-text responses of the “other”-category in the two previous study years (2021 and 2022); namely “I work in health care” and “To protect family or others”.

**Table 2:** Reasons for influenza vaccination

	Influenza season	2020/2021		2021/2022		2022/2023	
		*n*	%	*n*	%	*n*	%
**A: All vaccinated respondents in study sample**							
1	I belong to the RGs for severe influenza	156	26.2%	173	23.4%	172	22.8%
2	I want to protect myself against influenza	215	36.1%	304	41.2%	300	39.7%
3	The vaccine was easily accessible in the pharmacies	1	0.2%	6	0.8%	6	0.8%
4	Because of the COVID-19 pandemic	37	6.2%	13	1.8%	14	1.9%
5	The vaccine was free/nearly free this year	2	0.3%	3	0.4%	0	0.0%
6	Because of a stronger recommendation this year	8	1.3%	10	1.4%	16	2.1%
7	Due to increased media coverage	0	0.0%	4	0.5%	4	0.5%
8	My GP/other health professional recommended it	58	9.7%	75	10.2%	51	6.8%
9	I was offered the vaccine at work	92	15.4%	134	18.2%	51	6.8%
10	I work in health care	10	1.7%	2	0.3%	90	11.9%
11	To protect family members/others	11	1.8%	6	0.8%	38	5.0%
12	Other	5	0.8%	7	0.9%	13	1.7%
	*Item missing*	1	0.2%	1	0.1%	0	0.0%
	*Sum*	596	100%	738	100%	755	100%
**B: Vaccinated individuals belonging to the risk group (RG)**							
1	I belong to the RGs for severe influenza	135	37.8%	159	36.5%	155	35.7%
2	I want to protect myself against influenza	131	36.7%	165	37.8%	182	41.9%
3	The vaccine was easily accessible in the pharmacies	0	0.0%	3	0.7%	4	0.9%
4	Because of the COVID-19 pandemic	18	5.0%	6	1.4%	7	1.6%
5	The vaccine was free/nearly free this year	1	0.3%	1	0.2%	0	0.0%
6	Because of a stronger recommendation this year	4	1.1%	7	1.6%	9	2.1%
7	Due to increased media coverage	0	0.0%	3	0.7%	4	0.9%
8	My GP/other health professional recommended it	34	9.5%	54	12.4%	30	6.9%
9	I was offered the vaccine at work	20	5.6%	29	6.7%	11	2.5%
10	I work in health care	3	0.8%	1	0.2%	19	4.4%
11	To protect family members/others	6	1.7%	0	0.0%	10	2.3%
12	Other	4	1.1%	4	1.6%	3	0.7%
	*Item missing*	1	0.3%	1	0.2%	*0*	*0*
	*Sum*	357	100%	433	100%	434	100%
**C: Vaccinated health care workers (HCW)**							
1	I belong to the RGs for severe influenza	14	11.1%	6	5.0%	11	8.3%
2	I want to protect myself against influenza	25	19.8%	30	25.0%	20	15.2%
4	Because of the COVID-19 pandemic	4	3.2%	1	0.8%	2	1.5%
6	Because of a stronger recommendation this year	1	0.8%	0	0.0%	0	0.0%
8	My GP/other health professional recommended it	15	11.9%	13	10.8%	3	2.3%
9	I was offered the vaccine at work	57	45.2%	67	55.8%	9	6.8%
10	I work in health care	9	7.1%	2	1.7%	83	62.9%
11	To protect family members/others	0	0.0%	1	0.8%	3	2.3%
12	Other	1	0.8%	0	0.0%	1	0.8%
	*Sum*	126	100%	120	100%	132	100%

Table 2 presents reasons for influenza vaccination based on the question “State the most important reason why you got vaccinated”. Results are presented in absolute numbers and percent and are reported by influenza season for (A) all vaccinated respondents in the study sample, (B) vaccinated individuals belonging to the risk group (RG), and (C) vaccinated health care workers (HCWs), respectively. Data from Statistics Norway’s T&V-survey, Q2 & Q3, 2020 to 2023

Note that sample B and C are subsamples of sample A. HCWs with concurrent RG status are represented in all 3 samples (*n* = 26 individuals in 2020/21, *n* = 35 in 2021/22, and *n* = 39 in 2022/23. Missing respondents included 1 “do not know” in 2020/21, and 1 that refused to answer in 2021/22. Note also that there are fewer categories reported for HCWs because no HCW reported availability in pharmacies (category 3), free vaccine [5] or increased media coverage [7] as their main reason for vaccination

### Data analysis

We calculated weighted proportions for influenza vaccination coverage in the general sample, as well as for the RGs and HCWs, as part of the descriptive analysis of the sample. The weighting variable is generated by Statistics Norway to account for known non-response bias and/or underrepresentation in the net sample compared to the general population by age, sex, county or educational attainment. Weighting is applied in analysis to reduce the impact of such underrepresentation in study results [20]. The categorical data on reasons for influenza vaccination and non-vaccination, including free-text answers categorised as “other”, were thereafter sorted, tabulated for vaccinees and non-vaccinees, and stratified by study year and vaccine indication – the whole sample compared to the RGs and the HCWs – to study whether reported drivers or barriers for vaccination showed patterns related to time or group affiliation. Analyses were performed in SPSS version 28.

## Results

Characteristics of the 9 705 respondents included in the net sample are presented in Table 3. We observed an increase in the proportion belonging to the oldest age group and the RGs during the study period. Self-reported influenza vaccination coverage also increased substantially over the study period, both in the years prior to, and during, the COVID-19 pandemic.

**Table 3:** Characteristics of the study participants

Influenza season	2015/16*		2019/20		2020/21		2021/22		2022/23	
Variables	N	%	N	%	N	%	N	%	N	%
Sample	1 108	*100*	2 448	*100*	2 133	*100*	1 964	*100*	2 052	*100*
*Men*	596	*50.7*	1 232	*50.8*	1 093	*50.8*	1 020	*50.4*	1 068	*51.1*
*Women*	512	*49.3*	1 216	*49.2*	1 040	*49.2*	944	*49.6*	984	*48.9*
Age group (years)										
*16–44*	521	*50.8*	1 127	*49.1*	982	*49.6*	867	*50.0*	905	*48.5*
*45–64*	384	*33.2*	866	*33.5*	748	*33.0*	676	*31.8*	745	*33.5*
*65–79*	203	*16.1*	455	*17.4*	403	*17.4*	421	*18.2*	402	*18.0*
≥ 1 chronic condition										
*Yes*	209	*18.3*	489	*20.0*	424	*20.0*	418	*20.1*	474	*22.8*
*No*	889	*81.7*	1 959	*80.0*	1 709	*80.0*	1 546	*79.9*	1 578	*77.2*
Risk group (RG)**										
*Yes*	344	*29.3*	787	*31.4*	681	*30.9*	668	*31.0*	718	*33.7*
*No*	764	*70.8*	1 661	*68.6*	1 452	*69.1*	1 296	*69.0*	1 334	*66.3*
Health care worker (HCW)										
*Yes*	124	*11.0*	293	*11.4*	240	*10.8*	209	*10.5*	246	*11.7*
*No*	984	*89.0*	2 155	*88.6*	1 893	*89.2*	1 755	*89.5*	1 806	*88.3*
Vaccination coverage										
*General sample*	115	*9.7*	584	*23.0*	596	*26.8*	738	*35.1*	755	*34.4*
*RG*	75	*20.6*	323	*39.7*	357	*50.8*	433	*63.1*	434	*58.3*
*HCW*	14	*10.4*	130	*44.1*	126	*50.5*	120	*55.6*	132	*50.1*

Data from Statistics Norway’s T&V-survey Q2 2016 and Q2 & Q3 2020 to 2023. Results are weighted

* The sample in 2015/16 covers only one of the quarterly surveys, Q2

** The risk group includes respondents reporting chronic conditions and/or individuals aged 65–79 years

### Non-vaccination

**Fig. 1:**
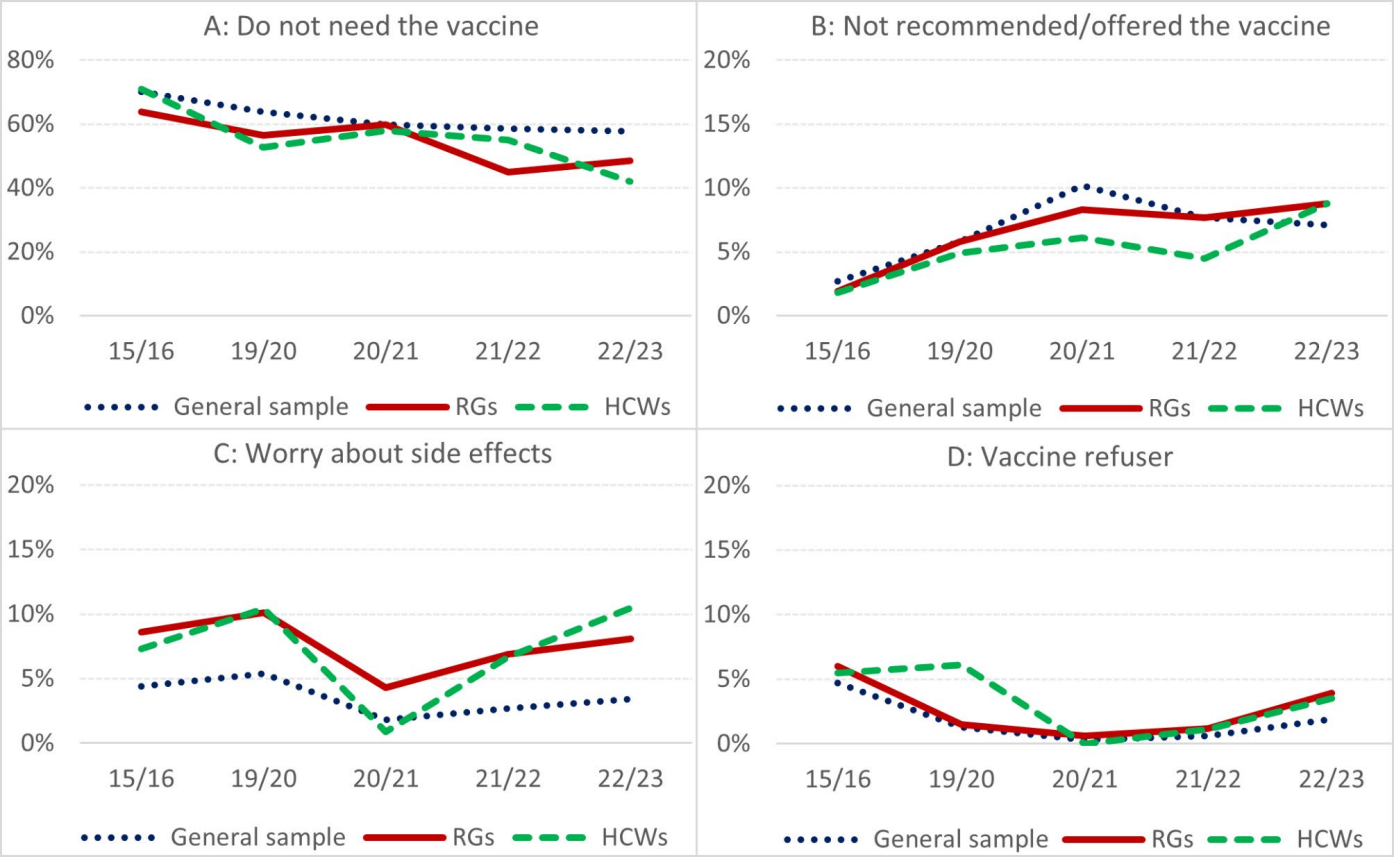
Reasons for non-vaccination by influenza season for the general sample, RGs and HCWs. Illustration of the proportion of the non-vaccinated respondents among the general sample, the risk groups (RGs) and health care workers (HCWs) reporting **A**) “Do not need the vaccine”, **B**) “Not recommended / offered the vaccine”, **C**) “Worry about side effects” or **D**) “Vaccine refuser” as their primary reason for non-vaccination each study year. *Data from Statistics Norway’s Travel & Vacation-survey*,* Q2 2016 and Q2 & Q3 2020 to 2023*

In all study years, among both the general sample, the RGs and the HCWs, “I do not need the vaccine” was the most frequently reported reason for influenza non-vaccination (Table 4; Fig. 1A). This was also the category that decreased the most from 2015/16 to 2022/23; from 70.1 to 57.8% among the general sample, from 63.8 to 48.6% among the RGs and, most notably, from 70.1 to 42.1% among the HCWs.

**Table 4:** Reasons for influenza non-vaccination

	Influenza season	2015/16		2019/2020		2020/2021		2021/2022		2022/2023	
		*n*	%	*n*	%	*n*	%	*n*	%	*n*	%
**A: All non-vaccinated respondents in study sample**											
**1**	Do not need the vaccine	681	70.1	1 190	63.8	921	59.9	717	58.5	750	57.8
**2**	No specific reason	98	10.1	315	16.9	343	22.3	292	23.8	278	21.4
**3**	Not recommended/offered the vaccine	26	2.7	108	5.8	157	10.2	94	7.7	92	7.1
**4**	Fear of doctors or injections	6	0.6	10	0.5	4	0.3	4	0.3	6	0.5
**5**	Prefer natural immunity	16	1.6	30	1.6	18	1.2	22	1.8	25	1.9
**6**	Do not believe in the vaccine	10	1.0	10	0.5	9	0.6	6	0.5	10	0.8
**7**	Worry about side effects	43	4.4	100	5.4	27	1.8	33	2.7	44	3.4
**8**	Vaccine refuser	46	4.7	24	1.3	4	0.3	7	0.6	25	1.9
**9**	Other	45	4.7	75	4.0	49	3.2	51	4.1	63	4.9
	*Item missing*	*0*	*0.0*	*2*	*0.1*	*5*	*0.3*	*0*	*0.0*	*4*	*0.3*
	*Sum*	*971*	*100*	*1 864*	*100*	*1 537*	*100*	*1 226*	*100*	*1 297*	*100*
**B: Non-vaccinated individuals belonging to the risk groups (RGs)**											
**1**	Do not need the vaccine	171	63.8	262	56.5	194	59.9	111	44.9	138	48.6
**2**	No specific reason	24	9.0	74	15.9	67	20.7	64	25.9	59	20.8
**3**	Not recommended/offered the vaccine	5	1.9	27	5.8	27	8.3	19	7.7	25	8.8
**4**	Fear of doctors or injections	2	0.7	5	1.1	1	0.3	0	0.0	1	0.4
**5**	Prefer natural immunity	7	2.6	12	2.6	2	0.6	11	4.5	10	3.5
**6**	Do not believe in the vaccine	5	1.9	4	0.9	5	1.5	1	0.4	4	1.4
**7**	Worry about side effects	23	8.6	47	10.1	14	4.3	17	6.9	23	8.1
**8**	Vaccine refuser	16	6.0	7	1.5	2	0.6	3	1.2	11	3.9
**9**	Other	15	5.6	26	5.6	10	3.1	21	8.5	13	4.6
	*Item missing*	*0*	*0.0*	*0*	*0.0*	*2*	*0.6*	*0*	*0.0*	*0*	*0.0*
	*Sum*	*268*	*100*	*464*	*100*	*324*	*100*	*247*	*100*	*284*	*100*
**C: Non-vaccinated health care workers (HCWs)**											
**1**	Do not need the vaccine	78	70.9	86	52.8	66	57.9	49	55.1	48	42.1
**2**	No specific reason	7	6.4	20	12.3	24	21.1	20	22.5	20	17.5
**3**	Not recommended/offered the vaccine	2	1.8	8	4.9	7	6.1	4	4.5	10	8.8
**4**	Fear of doctors or injections	1	0.9	0	0.0	1	0.9	0	0.0	0	0.0
**5**	Prefer natural immunity	0	0	6	3.7	5	4.4	2	2.2	3	2.6
**6**	Do not believe in the vaccine	2	1.8	2	1.2	1	0.9	0	0.0	2	1.8
**7**	Worry about side effects	8	7.3	17	10.4	1	0.9	6	6.7	12	10.5
**8**	Vaccine refuser	6	5.5	10	6.1	0	0.0	1	1.1	4	3.5
**9**	Other	6	5.5	14	8.6	9	7.9	7	7.9	15	13.2
	*Sum*	*110*	*100*	*163*	*100*	*114*	*100*	*89*	*100*	*114*	*100*

Table 4 presents reasons for influenza non-vaccination based on the question “State the most important reason why you did not get vaccinated”. Results are presented in absolute numbers and percent and are reported by influenza season for (A) all non-vaccinated respondents in the study sample, (B) non-vaccinated individuals belonging to the risk group (RG), and (C) non-vaccinated health care workers (HCWs), respectively. Data from Statistics Norway’s T&V-survey, Q2 2016 & Q2-Q3 2020 to 2023. Note that sample B and C are subsamples of sample A. HCWs with concurrent RG status are represented in all 3 samples (*n* = 21 in 2015/16, *n* = 26 individuals in 2019/20, *n* = 21 in 2020/21, *n* = 10 in 2021/22, and *n* = 22 in 2022/23). Missing respondents included 2 vaccinated individuals that wrongfully were included in the sample in 2019/20, and a total of 9 individuals that refused to answer the question in study year 2020/21 and 2022/23

The second most frequent category was “No specific reason” in all study years among both the general sample and the RGs, and in all but 2015/16 among the HCWs (where “worry about side effects” came in second at 7.3%). This was also the category that increased the most over time, at about 11% points from 2015/16 to 2022/23 in all three subsamples.

“Not recommended/offered the vaccine” also increased over time, especially among the target groups RGs and HCWs (Table 4; Fig. 1B), and lack of a recommendation was the third most frequent reason for non-vaccination among the general sample from 2019/20 to 2022/23, and among the RGs from 2020/21 to 2022/23.

The proportion reporting potential side effects as their main reason for non-vaccination, captured in the category “Worry about side effects”, fluctuated over the study period (Table 4; Fig. 1C). The proportion increased in all subsamples from 2015/16 to 2019/20, followed by a sharp decrease in 2020/21 before it increased once again in 2021/22 and 2022/23. While this pattern holds true for all groups, it was more pronounced among the RGs and especially the HCWs. It was also among the HCWs we observed both the highest (10.5% in 2022/23) and the lowest (0.9% in 2020/21) proportion for “worry about side effects” over the study period, and it was the second (2015/16) or third (2019/20, 2021/22 to 2022/23) most frequent category among HCWs in 4 out of 5 influenza seasons.

The proportion that was categorised as “vaccine refuser” was highest in the first study year, when it accounted for about 5–6% of the respondents in each subsample, before it fell to a negligible percentage of 0-0.6% during the COVID-19-pandemic, ending at 2–4% in 2022/23 (Fig. 1D).

The least frequent categories of all study years and groups were, at < 0.5% “Too expensive”, at < 1% “Fear of doctors or injections”, at < 2% “Do not believe in the vaccine”, and at < 5% “Prefer natural immunity”.

### Vaccine acceptance

**Fig. 2:**
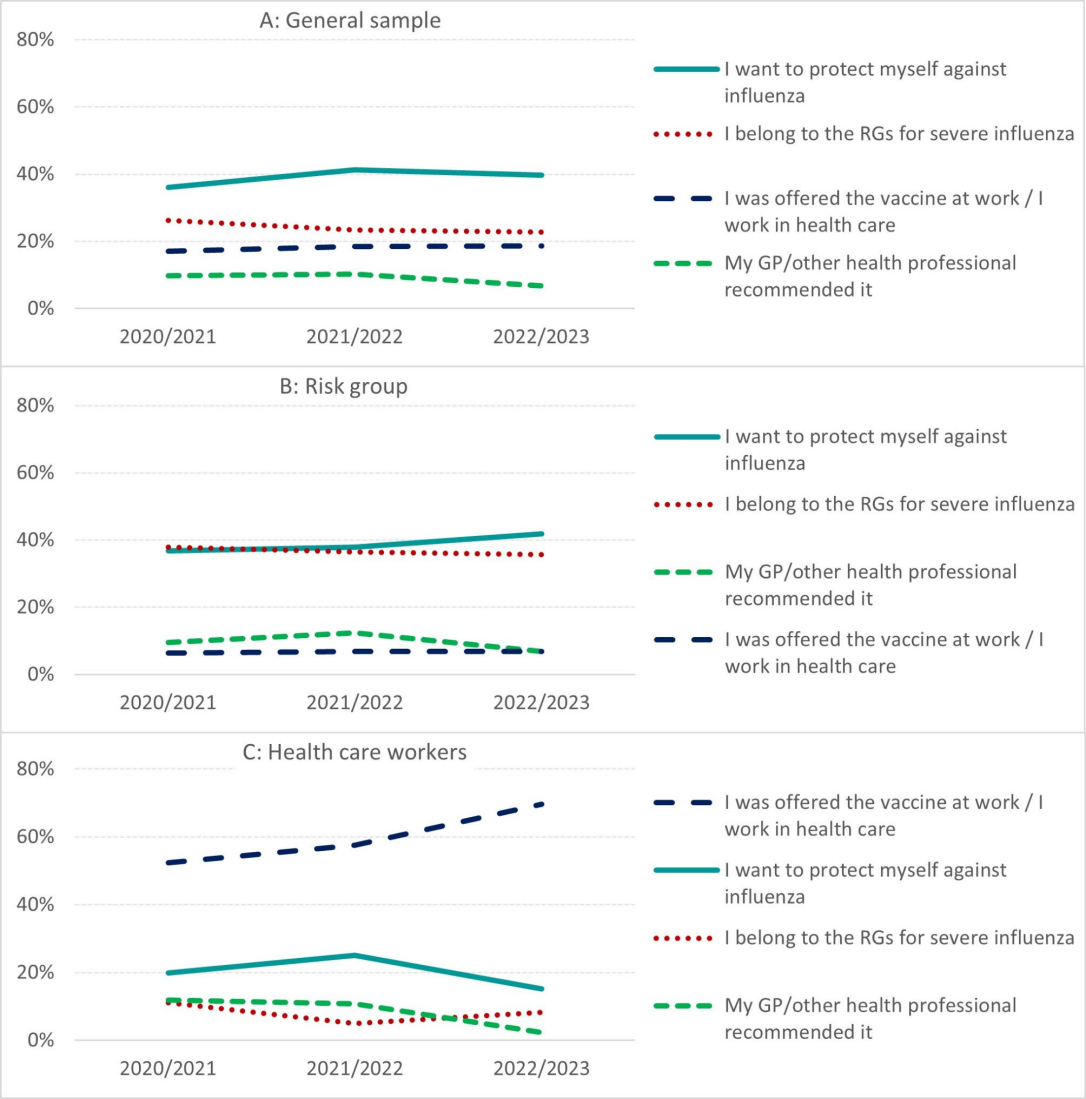
Reasons for vaccination by influenza season for the general sample, RGs and HCWs. Illustration of the four most frequent responses to the question “State the most important reason why you got vaccinated” by influenza season, among vaccinated respondents belonging to **A**) the general sample, **B**) the risk groups (RGs) and **C**) health care workers (HCWs), respectively. *Data from Statistics Norway’s T&V-survey*,* Q2 & Q3*,* 2020 to 2023*

There were notable differences in the most frequently stated reasons for influenza vaccination between the subsamples. In the general sample we observed the same pattern in all study years (Table 2A, Fig. 2A). Between 36.1 and 41.2% reported their primary reason as “I want to protect myself against influenza”, followed by 22.8–26.2% which stated that “I belong to the RGs for severe influenza”. The third most frequent answer was “I was offered the vaccine at work” in 2020/21 (15.4%) and 2021/22 (18.2%), while the category “I work in health care” came in third (at 11.9%) when it was introduced in 2022/23 (Table 2). When combined, the two categories on workplace vaccination averaged at about 18% of the respondents as the third most frequent reason each study year. Lastly, the fourth most frequent answer (at 9.7–6.8%), was “My GP/other health professional recommended it”.

We observed a similar pattern among the RGs, where “I want to protect myself against influenza” were only slightly more frequent than “I belong to the RGs for influenza” in all study years, varying between 36.7 to 41.9% for the former and between 35.6 to 37.8% for the latter (Table 2B, Fig. 2B). The third most frequent category, varying from 6.9–12.4% was “My GP/other health professional recommended it”. “I was offered the vaccine at work” came fourth in 2020/21 (5.6%) and 2021/22 (6.7%), while “I work in health care” came fourth in 2022/23 (4.4%). When combined, the two categories for workplace vaccination came in fourth in 2020/21 and 2021/22, while it shared third place with “My GP/other health professional recommended it in 2022/23”.

The most frequent reason for vaccination among HCWs was “I was offered the vaccine at work” in 2020/21 (45.2%) and 2021/22 (55.8%), and “I work in health care” (62.9%) in 2022/23 (Table 2C, Fig. 2C). When combined, the two categories for workplace vaccination accounted for 52.3%, 57.5% and 69.7% each study year respectively ‒ which represents an increase of 17.4% points over the three-year study period. The second most frequent response, at 19.8% in 2020/21, 25.0% in 2021/22 and 15.2% in 2022/23, was “I want to protect myself against influenza”. “My GP/other health professional recommended it” came third in 2020/21 (11.9%) and 2021/22 (10.8%), before it fell to fourth in 2022/23 (2.3%), when it switched places with “I belong to the RGs for severe influenza” (11.1% in 2020/21, 5% in 2021/22, and 8.3% in 2022/23).

## Discussion

We studied the reason why respondents in the general population, RGs and HCWs did or did not get vaccinated against influenza, and how these reasons varied by group and over time. The study covers reasons for influenza non-vaccination in seasons 2015/16 and 2019/20 to 2022/23, and reasons for vaccination in seasons 2020/21 to 2022/23. While the data have not been published previously, they have been used consecutively to inform the communication strategies of the Norwegian National Influenza Immunisation Programme [11].

The years covered in this study represents a period of increased influenza vaccine communication towards the public and HCWs from the health authorities, and an increased focus on vaccine availability and -coverage among RGs and HCWs towards the health services ‒ followed by the massive vaccine media coverage related to the COVID-19 pandemic starting in spring 2020. As presented in Table 3 and previously described, the measures implemented resulted in a substantial increase in influenza vaccination coverage [11] ‒ coinciding with an increase in influenza vaccine confidence [19] ‒ with a boost during the pandemic response that was seen also in a number of other countries [15, 22], and a slight reduction in the last study season.

### Reasons for non-vaccination

Past studies have found that common reasons for influenza non-vaccination include no perception of the need for vaccination, both in the general population [23–26] and in RGs [23, 26–29]. Respondents in these studies said that they did not belong to or did not recognise that they belonged to a group recommended for vaccination [23–25, 29], that they did not consider influenza to be a serious disease [25, 27, 29], did not feel at risk [28], or felt that they had sufficient immunity to influenza [29]. Not getting a vaccine recommendation from a health professional is another frequent response [23, 25, 28, 29], as is previous negative experiences or concerns about side effects [23–26, 28, 29], doubts about the effectiveness of the vaccine [23–25, 28], logistic difficulties such as time, convenience or cost [23, 24], no specific reason/not getting around to it [24, 26, 27], a lack of information [25] or a lack of trust in the vaccine/vaccines in general [23, 27].

Variations of the theme of no need for vaccination are common also in studies on HCWs, either because they question the indication (doubts of the vaccine’s ability to reduce nosocomial transmission) [30, 31], or the vaccine’s effectiveness [30, 32–34], express a low risk perception for influenza [31, 33, 35, 36] or feel that they are immune [30, 31, 36]. Convenience is also of importance [37]. Worry about side effects [30, 31, 33, 35–38] and related issues of vaccine safety and trust in the vaccine are frequently mentioned [31, 32, 34, 39], including misconceptions such as the idea that the vaccine may cause influenza [32, 33, 35, 37, 40].

The reasons for influenza non-vaccination frequently reported in the current study is in line with these previous results. The response patterns were similar among all three subsamples. The category “I do not need the vaccine”, including responses expressing that the respondents felt they were in good health, seldom got sick, or that they were confident that they could handle an influenza episode, were by far the most frequent response. This was followed by “no specific reason”, and thereafter “not recommended/offered the vaccine”, “worry about side effects” and “vaccine refuser”.

Although “I do not need the vaccine” clearly was the most frequent answer in all groups and study years, we also found that this category decreased substantially over the study period, both before and during the COVID-19-pandemic. While the pandemic clearly has impacted public opinion regarding vaccines, we therefore find it probable that the observed reduction is at least in part due to the aforementioned influenza campaigns focusing on availability and education. These campaigns specifically targeted HCWs and sought to strengthen their advocacy skills for the influenza vaccine, hoping for a “ripples in a pond”-effect [11, 15].

While the category “no need” implies that the respondent has formed an opinion as to whether influenza vaccination is relevant for him or her, the second most frequent category of “no specific reason” ‒ which increased year-by-year in all subsamples from 2015/16 until 2021/22 before a downwards turn in 2022/23 ‒ implies the opposite. One might argue that this category simply is an expression of influenza not being on people’s minds ‒ of complacency towards the risk of contracting influenza or of influenza as a potentially serious disease ‒ or even a sentiment related to vaccine fatigue [41].

“Not recommended/offered the vaccine” was the third most frequent response overall, and it also increased over the study period. This increase could partly result from an increasing awareness of the vaccine recommendations in the years leading up to and during the COVID-19 pandemic. However, several responses in the 2020/21 influenza season also point to the fact that influenza vaccine demand exceeded supply in this first pandemic winter, so that the available vaccine doses were reserved for the target groups, and in some municipalities just the RGs or vulnerable patient groups. Not receiving a recommendation under these circumstances could therefore be perceived as not being prioritised for vaccination.

While the three most common reasons for influenza non-vaccination in this study might be seen as expressions of no or low engagement with personal influenza vaccination choices, the last two categories of size point to either greater reluctance, as in “worry about side effects”, or to outright refusal, as in “vaccine refuser”. We observed fluctuations over time for these categories also, both before and under the COVID-19 pandemic. We can only speculate regarding the drivers behind the observed increase from 2015/16 to 2019/20, followed by a drop towards very modest levels in 2020/21. However, it is tempting to see the increase in reluctance towards the influenza vaccine in the years prior to the COVID-19 pandemic as a reaction to increased influenza vaccine communication in a period of, overall, relatively mild influenza seasons not long after a much-debated mass vaccination event in relation to the influenza pandemic in 2009. This in contrast to the drop in 2020/21 which might be an expression of a shift in the risk/benefit assessment for influenza vaccines, resulting from an increased risk awareness of infectious disease in combination with a heightened sense of societal responsibility in an active crisis. The acute drop in worry about side effects and vaccine refusal for the influenza vaccine could even be related to the more pronounced side effects experienced by many after the widespread COVID-19 vaccination, and the comparatively lesser side effects of the well-known influenza vaccine. Nonetheless, the recent increase in the last influenza seasons indicate that there is a vaccine-related unease in parts of the population that needs to be addressed in years to come, and future studies should consider if there are demographic patterns in the distribution of common reasons for non-vaccination that mirrors the observed educational pattern in general influenza vaccine confidence [19].

### Reasons for influenza vaccination

We found that the top four reasons for influenza vaccine acceptance were the same in all subsamples, but that their inwards ranking varied between the groups. In the general sample we observed the same stepwise pattern in all study years; protection against influenza was followed by belonging to a RG and workplace vaccination, and lastly recommendation from a health professional. Protection against influenza and belonging to a RG were also the most common reasons among the RGs ‒ whilst receiving a recommendation was slightly more frequent than work-based vaccination. As we surmise that the category of belonging to a RG is also related to protection against disease, either directly or through an awareness of increased risk of severe disease, we conclude that the desire to protect oneself against influenza clearly was the most common reason for vaccination in these two subsamples, in line with several earlier studies among both the general population [24, 25, 40, 42] and among RGs [13, 42].

The finding that a personal recommendation from a GP or another trusted health professional is important for the decision to vaccinate is previously shown in studies among both the general adult population [13, 23, 25, 40], among RGs in general [23, 28], and among the elderly [23–25]. We also observed that a personal recommendation was among the most frequent reasons for vaccination - just as lack of such a recommendation was among the frequent reasons for non-vaccination. This is in line with earlier studies that have looked at the role of a personal recommendation as both barrier and facilitator for vaccination [25, 28], further strengthening the knowledge that health professionals’ personal advice may influence their patients’ vaccination coverage directly.

When examining reasons for influenza vaccine acceptance among HCWs, common reasons for taking the influenza vaccine have been found to include wanting to protect self, family members, co-workers and patients [22, 31, 33, 35, 36, 43–45], easy access to free vaccination services [35, 43, 45], as well as workplace recommendation and peer pressure [31, 35, 36, 43–45]. While protection against influenza was the second most frequent reason for vaccination among HCWs in our study, and being in a RG or getting a recommendation was mentioned, work-based vaccination, covering the categories “I work in health care” and “I was offered the vaccine at work”, was clearly the most frequently cited reason at on average 60% of the responses. While these categories probably hold aspects of both ease of access, workplace norms and expectations, as well as a sense of obligation and wanting to protect both oneself and others, it would be interesting to explore the HCWs reasoning further in future studies.

### Strengths and limitations

This study is part of a well-designed population telephone survey that collects data for various national statistics on a regular basis. The sampling frame is the Norwegian National Register where every citizen has a unique identifier, and the survey has a large sample and high response rates. Statistics Norway also publishes estimates for over- and underrepresentation of various groups. We are therefore aware that individuals in the younger age groups (25–44 years) and individuals of no or low education are underrepresented in the sample compared to the general population [20], resulting in higher estimates of influenza vaccination coverage in the survey compared to register data. The difference amounts to approximately 5% points for estimates of the general population and the RGs in the last two study seasons (2021/22 and 2022/23), where registry data are available and reliable. The difference is higher for HCWs (10–12% points) - but it must also be noted that it is difficult to estimate accurate vaccination coverage for this group via registry data in Norway, as the HCW population tends to be overestimated in the registries and vaccinations are underreported.

We are fortunate to have data from several study seasons, enabling us to observe and discuss national variations over time, including potential impacts of the recent COVID-19 pandemic response. We have found these low-cost survey items to be useful in our continuous work to monitor influenza vaccine hesitancy and increase and maintain influenza vaccination coverage on a limited budget. However, as this is a study of cross-sectional design, we are unable to draw conclusions regarding cause and effect. Furthermore, as this questionnaire and the categorisation system were developed by the authors with the intent to directly inform the communication strategies and the priorities of the national influenza immunisation programme, the possibility of direct comparison with other countries is limited. Note also that the respondents in this study were asked, over the phone, to give in their own words their “most important reason” for vaccination/non-vaccination, whereas most previous studies – often written questionnaires where the respondents are presented with several options ‒ allow for several reasons, and thus several categories with higher percentages compared to the results of our study. While several factors influence personal vaccination/non-vaccination choices, we are therefore only able to discuss the factors reported by each respondent as their most important incentive.

## Conclusions

In this study we found that common reasons for influenza non-vaccination differs but little between the general population, individuals in the RGs for severe influenza or individuals working as HCWs with patient contact; the clearly most cited reasons are “no need” for the vaccine and “no specific reason”, followed by “no recommendation” and expressions of worry regarding the vaccine or vaccine refusal.

On the other hand, the most important reasons for influenza vaccine acceptance do vary between subsamples. The desire for protection against influenza or a statement of belonging to the RGs are clearly the most frequent among the general sample and the RGs, whilst this category ranks second among the HCWs, for whom categories related to work-based vaccination or a reference to their role as health professionals are most cited.

While we have seen positive developments over the study period, such as an increase in influenza vaccination coverage and a reduction in the proportion reporting that they do not need the vaccine, our results indicate a need to keep up a solid knowledge-based vaccine communication to increase awareness and maintain – or preferably increase – influenza vaccine confidence.

Futures studies should explore HCWs viewpoints regarding workplace vaccination, and study reasons for influenza vaccination/non-vaccination in relation to sociodemographic factors that are known to affect vaccine coverage and confidence in Norway, such as age or education. Insight into such patterns in the “why” could lead to targeted measures to improve vaccine access, knowledge, or trust in currently underserved groups.

## Data Availability

The datasets supporting the findings of this article are available from the Survey Bank repository at Sikt – Norwegian Agency for Shared Services in Education and Research, [https://sikt.no/en/tjenester/finn-data/survey-bank].

## Abbreviations

HCW: Health care worker
RG: Risk group
T&V-survey: Statistics Norway’s Travel & Vacation survey
GP: General Practitioner
Q2 / Q3: Refers to the second /third quarter of the year

## References

[1] Lafond KE, Porter RM, Whaley MJ, Suizan Z, Ran Z, Aleem MA et al. Global burden of influenza-associated lower respiratory tract infections and hospitalizations among adults: a systematic review and meta-analysis. PLoS Med. 2021;18(3). 10.1371/journal.pmed.1003550 PMC7959367 33647033

[2] Cozza V, Campbell H, Chang HH, Iuliano AD, Paget J, Patel NN, et al. Global Seasonal Influenza Mortality estimates: a comparison of 3 different approaches. Am J Epidemiol. 2020;190(5):718–27. 10.1093/aje/kwaa196 PMC8218989 32914184

[3] Paget J, Staadegaard L, Wang X, Li Y, van Pomeren T, van Summeren J, et al. Global and national influenza-associated hospitalisation rates: estimates for 40 countries and administrative regions. J Glob Health. 2023;13:04003. 36701368 10.7189/jogh.13.04003 PMC9879557

[4] Barberis I, Myles P, Ault SK, Bragazzi NL, Martini M. History and evolution of influenza control through vaccination: from the first monovalent vaccine to universal vaccines. J Prev Med Hyg. 2016;57(3):E115–20. 27980374 PMC5139605

[5] Palache A, Rockman S, Taylor B, Akcay M, Billington JK, Barbosa P, et al. Vaccine complacency and dose distribution inequities limit the benefits of seasonal influenza vaccination, despite a positive trend in use. Vaccine. 2021;39(41):6081–7. 34521551 10.1016/j.vaccine.2021.08.097 PMC8433505

[6] World Health Organization. Vaccines against influenza: WHO position paper – may 2022. Wkly Epidemiol Rec. 2022;97(19):185–208.

[7] World Health Assembly. Fifty-sixth World Health Assembly, Geneva, 19–28 May 2003: resolutions and decisions, annexes - Prevention and control of influenza pandemics and annual epidemics. 2003 https://apps.who.int/iris/bitstream/handle/10665/259836/WHA56-2003-REC1-eng.pdf?sequence=1&isAllowed=y

[8] The Council of the European Union. Council recommendation of 22 December 2009 on seasonal influenza vaccination. Official Journal of the European Union [Internet]. 2009. https://ec.europa.eu/health/sites/health/files/vaccination/docs/seasonflu_staffwd2014_en.pdf

[9] Folkehelseinstituttet. Influensavaksinasjonsprogrammet. Folkehelseinstituttet 2023 [updated 30.06.2023]. https://www.fhi.no/va/vaksinasjonsveilederen-for-helsepersonell/vaksinasjon/influensa/?term=.

[10] Klüwer B, Rydland KM, Laake I, Todd M, Juvet LK, Mamelund S-E. Influenza risk groups in Norway by education and employment status. Scand J Public Health. 2021;50(6):756–64. 34930055 10.1177/14034948211060635 PMC9361423

[11] Klüwer B, Rydland KM, Nybru Gleditsch R, Mamelund SE, Laake I. Social and demographic patterns of influenza vaccination coverage in Norway, influenza seasons 2014/15 to 2020/21. Vaccine. 2023;41(6):1239–46. 36639272 10.1016/j.vaccine.2023.01.013

[12] Endrich MM, Blank PR, Szucs TD. Influenza vaccination uptake and socioeconomic determinants in 11 European countries. Vaccine. 2009;27(30):4018–24. 19389442 10.1016/j.vaccine.2009.04.029

[13] Yeung MPS, Lam FLY, Coker R. Factors associated with the uptake of seasonal influenza vaccination in adults: a systematic review. J Public Health (Oxf). 2016;38(4):746–53. 28158550 10.1093/pubmed/fdv194

[14] Anastasiou OE, Heger D. Understanding the Influence of Individual and systemic factors on Vaccination Take-Up in European citizens aged 55 or older. Vaccines. 2021;9(2):169. 33671437 10.3390/vaccines9020169 PMC7922776

[15] Welch VL, Metcalf T, Macey R, Markus K, Sears AJ, Enstone A, et al. Understanding the barriers and attitudes toward Influenza Vaccine Uptake in the Adult General Population: a Rapid Review. Vaccines. 2023;11(1):180. 36680024 10.3390/vaccines11010180 PMC9861815

[16] Bragstad K, Paulsen TH, Seppälä EM, Tønnessen R, Klüwer B, Rydland KM, et al. Influenza virological and epidemiological season report, October 2022. Oslo, Norway: Norwegian Institute of Public Health; 2022.

[17] Brewer NT, Chapman GB, Rothman AJ, Leask J, Kempe A. Increasing vaccination: putting Psychological Science Into Action. Psychological science in the public interest: a. J Am Psychol Soc. 2017;18(3):149–207. 10.1177/1529100618760521 29611455

[18] Steens A, Stefanoff P, Daae A, Vestrheim DF, Riise Bergsaker MA. High overall confidence in childhood vaccination in Norway, slightly lower among the unemployed and those with a lower level of education. Vaccine. 2020. 10.1016/j.vaccine.2020.05.011 32448621

[19] Klüwer B, Gleditsch R, Rydland KM, Mamelund S-E, Laake I. Higher educational attainment associated with higher confidence in influenza vaccination in Norway. Vaccine. 2024;42(11):2837–47. 38519343 10.1016/j.vaccine.2024.03.049

[20] Statistics Norway. Reise- og ferieundersøkelsen. Dokumentasjon [Travel & Vacation Survey, Documentation; In Norwegian] Oslo: Statistics Norway. 2023 [ https://www.ssb.no/transport-og-reiseliv/reise-og-ferieundersokelsen.dokumentasjon

[21] MacDonald NE, Sage Working Group on Vaccine Hesitancy. Vaccine hesitancy: definition, scope and determinants. Vaccine. 2015;33(34):4161–4. 25896383 10.1016/j.vaccine.2015.04.036

[22] Guerrero-Soler M, Gras-Valenti P, Platas-Abenza G, Sánchez-Payá J, Sanjuan-Quiles Á, Chico-Sánchez P, et al. Impact of the COVID-19 pandemic on Influenza Vaccination Coverage of Healthcare Personnel in Alicante, Spain. Vaccines. 2024;12(4):370. 38675752 10.3390/vaccines12040370 PMC11055171

[23] Farmanara N, Sherrard L, Dube E, Gilbert NL. Determinants of non-vaccination against seasonal influenza in Canadian adults: findings from the 2015–2016 Influenza Immunization Coverage Survey. Can J Public Health. 2018;109(3):369–78. 29981075 10.17269/s41997-018-0018-9 PMC6153712

[24] Abbas KM, Kang GJ, Chen D, Werre SR, Marathe A. Demographics, perceptions, and socioeconomic factors affecting influenza vaccination among adults in the United States. Peerj. 2018;6:e5171. 30013841 10.7717/peerj.5171 PMC6047499

[25] Prada-García C, Fernández-Espinilla V, Hernán-García C, Sanz-Muñoz I, Martínez-Olmos J, Eiros JM et al. Attitudes, perceptions and practices of Influenza Vaccination in the Adult Population: results of a cross-sectional survey in Spain. Int J Environ Res Public Health. 2022;19(17). 10.3390/ijerph191711139 PMC9518428 36078854

[26] Roy M, Sherrard L, Dube E, Gilbert NL. Determinants of non-vaccination against seasonal influenza. Health Rep. 2018;29(10):12–22. 30329145

[27] Bodeker B, Remschmidt C, Schmich P, Wichmann O. Why are older adults and individuals with underlying chronic diseases in Germany not vaccinated against flu? A population-based study. BMC Public Health. 2015;15:618. 26148480 10.1186/s12889-015-1970-4 PMC4492002

[28] Bertoldo G, Pesce A, Pepe A, Pelullo CP, Di Giuseppe G, Collaborative Working G. Seasonal influenza: knowledge, attitude and vaccine uptake among adults with chronic conditions in Italy. PLoS ONE [Electronic Resource]. 2019;14(5):e0215978. 31042752 10.1371/journal.pone.0215978 PMC6493755

[29] Kroneman M, van Essen GA, John Paget W. Influenza vaccination coverage and reasons to refrain among high-risk persons in four European countries. Vaccine. 2006;24(5):622–8. 16169638 10.1016/j.vaccine.2005.08.040

[30] Hagemeister MH, Stock NK, Ludwig T, Heuschmann P, Vogel U. Self-reported influenza vaccination rates and attitudes towards vaccination among health care workers: results of a survey in a German university hospital. Public Health. 2018;154:102–9. 29220709 10.1016/j.puhe.2017.10.027

[31] Lorenc T, Marshall D, Wright K, Sutcliffe K, Sowden A. Seasonal influenza vaccination of healthcare workers: systematic review of qualitative evidence. BMC Health Serv Res. 2017;17(1):732. 29141619 10.1186/s12913-017-2703-4 PMC5688738

[32] Halpin C, Reid B. Attitudes and beliefs of healthcare workers about influenza vaccination. Nurs Older People. 2019;31(2):32–9. 31468782 10.7748/nop.2019.e1154

[33] Lehmann BA, Ruiter RA, Wicker S, van Dam D, Kok G. I don’t see an added value for myself: a qualitative study exploring the social cognitive variables associated with influenza vaccination of Belgian, Dutch and German healthcare personnel. BMC Public Health. 2014;14:407. 24775096 10.1186/1471-2458-14-407 PMC4021212

[34] Pavlovic D, Sahoo P, Larson HJ, Karafillakis E. Factors influencing healthcare professionals’ confidence in vaccination in Europe: a literature review. Hum Vaccin Immunother. 2022;18(1):2041360. 35290160 10.1080/21645515.2022.2041360 PMC9009961

[35] Boey L, Bral C, Roelants M, De Schryver A, Godderis L, Hoppenbrouwers K, et al. Attitudes, believes, determinants and organisational barriers behind the low seasonal influenza vaccination uptake in healthcare workers - a cross-sectional survey. Vaccine. 2018;36(23):3351–8. 29716777 10.1016/j.vaccine.2018.04.044

[36] Lindvig SO, Larsen L. Uptake of and attitudes towards influenza vaccination among Danish hospital healthcare workers. Dan Med J. 2021;68(3):16. 33660606

[37] Neufeind J, Wenchel R, Boedeker B, Wicker S, Wichmann O. Monitoring influenza vaccination coverage and acceptance among health-care workers in German hospitals - results from three seasons. Hum Vaccin Immunother. 2021;17(3):664–72. 33124954 10.1080/21645515.2020.1801072 PMC7993141

[38] Colaprico C, Ricci E, Bongiovanni A, Imeshtari V, Barletta VI, Manai MV et al. Flu vaccination among Healthcare professionals in Times of COVID-19: knowledge, attitudes, and Behavior. Vaccines (Basel). 2022;10(8). 10.3390/vaccines10081341 PMC9414714 36016229

[39] Mytton OT, O’Moore EM, Sparkes T, Baxi R, Abid M. Knowledge, attitudes and beliefs of health care workers towards influenza vaccination. Occup Med. 2013;63(3):189–95. 10.1093/occmed/kqt002 23447033

[40] Trent MJ, Salmon DA, MacIntyre CR. Using the health belief model to identify barriers to seasonal influenza vaccination among Australian adults in 2019. Influenza Other Respir Viruses. 2021;15(5):678–87. 33586871 10.1111/irv.12843 PMC8404057

[41] Skyles TJ, Stevens HP, Obray AM, Jensen JL, Miner DS, Bodily RJ, et al. Changes in attitudes and barriers to Seasonal Influenza Vaccination from 2007 to 2023. J Community Health. 2024;49(2):207–17. 37697225 10.1007/s10900-023-01277-7

[42] Nowak GJ, Sheedy K, Bursey K, Smith TM, Basket M. Promoting influenza vaccination: insights from a qualitative meta-analysis of 14 years of influenza-related communications research by U.S. Centers for Disease Control and Prevention (CDC). Vaccine. 2015;33(24):2741–56. 25936726 10.1016/j.vaccine.2015.04.064 PMC5856146

[43] Hofmann F, Ferracin C, Marsh G, Dumas R. Influenza vaccination of healthcare workers: a literature review of attitudes and beliefs. Infection. 2006;34(3):142–7. 16804657 10.1007/s15010-006-5109-5

[44] Schmid P, Rauber D, Betsch C, Lidolt G, Denker ML. Barriers of Influenza Vaccination Intention and behavior - A systematic review of Influenza Vaccine Hesitancy, 2005–2016. PLoS ONE. 2017;12(1):e0170550. 28125629 10.1371/journal.pone.0170550 PMC5268454

[45] Bianchi FP, Stefanizzi P, Di Lorenzo A, De Waure C, Boccia S, Daleno A, et al. Attitudes toward influenza vaccination in healthcare workers in Italy: a systematic review and meta-analysis. Hum Vaccin Immunother. 2023;19(3):2265587. 37849235 10.1080/21645515.2023.2265587 PMC10586073

